# Assessment of Nutritional Knowledge, Dietary Habits and Nutritional Status of Cardiology Patients, Considering Differences Between Individuals with Hypertension and Atherosclerosis and Those Without These Conditions

**DOI:** 10.3390/nu17050754

**Published:** 2025-02-21

**Authors:** Anna-Maria Sapała, Wiktoria Staśkiewicz-Bartecka, Agata Kiciak, Marek Kardas

**Affiliations:** 1 Department of Dietetics and Food Science, Faculty of Science, Natural and Technical Sciences, Jan Długosz University in Częstochowa, al. Armii Krajowej 13/15, 42-200 Częstochowa, Poland; am.stelmach@ujd.edu.pl; 2 Department of Food Technology and Quality Assessment, School of Public Health in Bytom, Medical University of Silesia in Katowice, ul. Jordana 19, 41-808 Zabrze, Poland; wstaskiewicz@sum.edu.pl (W.S.-B.); mkardas@sum.edu.pl (M.K.)

**Keywords:** dietary habits, cardiac patients, hypertension, atherosclerosis, nutritional knowledge, nutritional status

## Abstract

Background/Objectives: Proper nutrition and correct habits and behaviours are crucial elements in the treatment and prevention of hypertension or atherosclerosis. This study aims to assess the nutritional knowledge, dietary habits, and nutritional status of cardiology patients, particularly those with hypertension and atherosclerosis. Methods: The study was conducted at St. Barbara Regional Specialized Hospital No. 5 in Sosnowiec from January to June 2021. It included 301 patients, 51.2% women (N = 154) and 48.8% men (N = 147), who were assessed for Body Mass Index, Nutritional Risk Score, and morphological and biochemical test results. Their knowledge and dietary habits were examined using a questionnaire and rating scale designed by the authors. While the study encompassed all cardiology patients, a subgroup analysis specifically examined individuals diagnosed with hypertension and/or atherosclerosis. Results: 80% of the respondents showed above-normal body weight, while there were no significant differences in the risk of malnutrition according to the Nutritional Risk Score. The diet analysis revealed insufficient intake of fruits, vegetables, legumes, dairy, whole grains, and fish, while the consumption of salty snacks, sweetened beverages, energy drinks, and alcohol was low. Analysis of morphology and biochemistry results showed no significant differences between patients with atherosclerosis, hypertension, and others. Conclusions: The study reveals insufficient nutritional knowledge and poor dietary habits among cardiology patients, highlighting the need for targeted education to improve dietary behaviours and reduce cardiovascular risks. Implementing nutrition-focused interventions in cardiology care could enhance patient outcomes. Future research should explore the long-term effects of dietary education and identify effective strategies for behavioural change in this population.

## 1. Introduction

Scientific and technological advances and the general development of civilisation have improved the population’s health consciousness. Unfortunately, this development is also accompanied by numerous diseases, and one of the biggest problems is metabolic syndrome, which includes obesity, atherosclerosis, hypertension as well as type 2 diabetes [1,2].

Arterial hypertension is one of the cardiovascular diseases that is usually characterised by primary elevated blood pressure. This means that the causes of the development of the disease are usually not clearly defined, but environmental and genetic factors are most commonly cited among them. It is often latent in nature, i.e., asymptomatic until complications occur. Symptoms of hypertension are not characteristic and include palpitations, headaches and general fatigue. The most characteristic symptom is a severe headache in the occipital region, accompanied by an aura. This phenomenon involves the simultaneous occurrence of other neurological disorders, such as speech, vision or consciousness and orientation disturbances, which most often occur in the morning. The most common complications of untreated hypertension are chronic renal failure, as well as so-called ‘higher’ cardiovascular disease, which may include ischaemic stroke or myocardial infarction [3,4,5].

According to the definition of the Polish Society of Hypertension (PTNT), we classify the disease when the systolic pressure value exceeds 140 mm Hg and the diastolic pressure value exceeds 90 mm Hg. However, it is important to note that these values apply to people who are not on pharmaceutical treatment. A special case is the malignant form of hypertension, in which the diastolic pressure reaches values of 120–140 mm Hg. It is extremely difficult to treat as it is accompanied by vascular damage to the retina and multi-organ failure, including heart failure [3,5,6,7,8].

It is estimated that approximately 1 billion people worldwide are affected by hypertension, and this number is steadily increasing and is expected to rise to as many as 1.5 billion by the end of 2025 [59 doc]. Normalisation of body weight and a well-balanced, rational diet, which includes, in particular, a reduction in the intake of saturated fatty acids and salt, as well as cessation of stimulant consumption, including alcohol and smoking, and increased physical activity will be preventive factors. In addition, these factors are non-pharmacological therapies, which, when hypertension is asymptomatic, can be a life-saving measure [3,5,9].

Dyslipidemia is a metabolic disorder characterised by abnormal plasma lipid and lipoprotein concentrations, which may be influenced by factors such as obesity, diet, and genetic predisposition [9]. In the diagnosis of metabolic syndrome, we distinguish between atherogenic dyslipidemia, which is characterised by triglyceride levels above 150 mg/dL, increased LDL cholesterol (Low-Density Lipoproteins) between 100 and 160 mg/dL (depending on cardiovascular risk level), and decreased HDL cholesterol (High-Density Lipoproteins) below 40 mg/dL in men and 50 mg/dL in women, according to the European Society of Cardiology (ESC) guidelines. Additionally, hypercholesterolemia is defined as total cholesterol levels above 200 mg/dL, often accompanied by an elevated LDL fraction [10,11,12,13].

Dyslipidaemias, like hypertension, can develop as latent diseases and only give the first symptoms in the form of dangerous cardiovascular disease. The result of abnormal cholesterol levels is damage to the walls of the blood vessels and, consequently, the formation of atherosclerotic foci and the development of atherosclerosis. The factors that cause the development of lipid disorders and the resulting diseases are divided into modifiable and non-modifiable ones. Non-modifiable factors include genetic predisposition, gender and age, while modifiable factors include body weight, physical activity and stimulants [13,14,15].

Proper nutrition and correct habits and behaviours are the most important elements in the treatment and prevention of hypertension or atherosclerosis. The metabolic syndrome is an increasing problem that includes obesity, and this is a result of inappropriate eating habits and insufficient physical activity [16,17]. Nutrition education and the implementation of appropriate habits is crucial to reduce the risk of complications caused by non-adherence to the diet [18].

The primary objective of our study was to assess knowledge and dietary habits and relate them to the nutritional status of patients hospitalised in the cardiology department, including those diagnosed with hypertension and/or atherosclerosis. The hypothesis was that patients diagnosed with these conditions would show differences in knowledge scores and eating habits, which would, in turn, affect their nutritional status compared to subjects without these conditions.

## 2. Materials and Methods

### 2.1. Study Design

The study was a cross-sectional observational study conducted in Poland, in Sosnowiec, at the St. Barbara Regional Hospital No. 5 in the cardiology department in 2021, during the months of January to June. Respondents were admitted to the hospital for cardiovascular complaints (I00-I99 in the International Classification of Diseases ICD-10). Approval for the analysis was obtained from the Bioethics Committee of the Silesian Medical University in Katowice, Poland, No. PCN/0022/KB/299/19/20, which was issued on 21 January 2020. Respondents who took part in the following study knew its purpose and expectations based on the Declaration of Helsinki. To participate in the study, written and verbal consent had to be completed.

### 2.2. Study Participants

The survey group consisted of 51.2% women (154 people) and 48.8% men (147 people), totalling 301 respondents residing in a cardiology department in a Polish hospital. To participate, respondents had to be at least 18 years old, registered in the Silesian province, diagnosed with cardiac disease, and hospitalised for at least three days. Additionally, they had to provide written consent to participate in the analysis.

Exclusion criteria included individuals with diagnosed mental disorders, advanced kidney or liver disease, oncological diseases, or other conditions that could significantly impair their ability to complete the questionnaire accurately. Additionally, incomplete responses led to exclusion from the analysis.

### 2.3. Research Tools

#### 2.3.1. Nutritional Status of Patients

Body mass index (BMI)—calculated from the formula:

(1) $$BMI=\frac{body\;weight\;\left[kg\right]}{({height[m])}^{2}}$$

The categorisation of nutritional status using BMI was conducted according to the World Health Organization (WHO). A BMI of <18.49 kg/m^2^ indicates underweight, while a BMI in the range of 18.5–24.99 kg/m^2^ is considered normal body weight. A BMI of 25–29.99 kg/m^2^ indicates overweight, whereas a BMI of 30 kg/m^2^ or higher is classified as obesity. Obesity is further divided into three categories: 1st-degree obesity (BMI up to 34.99 kg/m^2^), 2nd-degree obesity (BMI 35–39.99 kg/m^2^), and 3rd-degree obesity (BMI > 40 kg/m^2^) [19].

#### 2.3.2. NRS 2002 Scale

The NRS 2002 (Nutritional Risk Score) scale is an index used to assess patients’ nutritional status. It evaluates both nutritional status and disease severity. According to Kondrup et al. [2,3], this scale consists of three parts: assessment of the patient’s nutritional status (0–3 points), disease severity (0–3 points), and age (1 point for ≤70 years).

The maximum possible score is 7, and a patient receiving a score of 3 or more is classified as being at risk of malnutrition according to the NRS 2002. The nutrition scan score on this scale is determined by body mass index (BMI), weight loss over 1–3 months (≥5%), and the type and amount of food consumed in the past week. This is assessed through a nutritional interview with a dietitian prior to hospitalisation and compared with the patient’s usual food intake. The results are divided into four categories: 0–25%, 25–50%, 50–75%, and 100% of energy requirements [20,21]

#### 2.3.3. Analysis of Knowledge and Eating Habits

In order to collect all the necessary information from the respondents, the dietitian conducted individual nutritional interviews, through which they obtained information regarding the respondents’ age, place of residence, education, and lifestyle. A diagnostic survey method was used, along with a proprietary questionnaire that, in addition to a demographic section, included parts examining the patients’ nutritional knowledge and dietary habits, including the frequency of food consumption.

The first part of the questionnaire consisted of demographic questions: gender, age, education, diet used and diagnosed diseases. The questionnaire assessing the patients’ nutritional knowledge consisted of 19 questions, where only one answer was correct.

The third part of the study focused on the respondents’ eating habits and the frequency of consumption of specific food products, which was assessed using a food frequency questionnaire (FFQ).

The assessment of food frequency used a 6-point scale: several times a day, daily, several times a week, several times a month, once a month or less often, never or rarely. The FFQ was developed based on the dietary guidelines in force in Poland in 2011 for both women and men and was based on the recommendations of the Institute of Food and Nutrition (IŻŻ) in Warsaw.

The questionnaire on the frequency of consumption of selected foods was divided into eight sections: cereals and cereal products, milk and dairy products such as kefir, buttermilk, yoghurt, cheese, and eggs, meat, cold cuts, and fish, vegetables, which were also categorised into several groups, as well as grains, fruits, products that are sources of fat, beverages, sugar, honey and sweets.

To assess patients’ eating habits, a proprietary scoring scale was also used. Similar to the nutritional knowledge assessment, patients received 1 point for a correct answer and 0 points for an incorrect answer. The total number of points was then summed and presented as a percentage in relation to the maximum number of responses indicating the best eating habits. The same scale was used for evaluating both eating habits and respondents’ nutritional knowledge.

The validation process of the questionnaire consisted of several stages. In the first phase, a preliminary version of the research tool was developed, and the questions were verified by experts in the field of nutrition for their substantive accuracy and compliance with current dietary guidelines. Subsequently, a pilot study was conducted on a group of 30 respondents to assess the clarity and comprehensibility of the questions. The results of the pilot study were subjected to detailed analysis and discussion among experts, allowing for the identification of questions and answers that raised doubts. Based on the collected feedback, necessary corrections were made by eliminating ambiguities and refining the wording of the questions to enhance the precision and validity of the research tool.

#### 2.3.4. Biochemical Tests and Blood Count of Patients

The biochemical tests of all study participants were collected on an empty stomach, first thing in the morning, after a 12 h overnight fast. Blood samples were obtained via venipuncture and processed for analysis. The biochemical analyses were performed on serum samples, which were separated by centrifugation at 3000 rpm for 10 min. Haematological parameters, including WBC (leukocyte level), RBC (erythrocyte level), PLT (platelets), Hgb (haemoglobin), and Hct (haematocrit), were determined using an automated haematology analyser (e.g., Sysmex or equivalent). Biochemical tests included fasting glucose (FG), total cholesterol (TC), LDL cholesterol (LDL-C), HDL cholesterol (HDL-C), and triglycerides (TG), which were measured using an enzymatic colourimetric method. Electrolyte levels, including sodium (Na) and potassium (K), were determined using an ion-selective electrode method (ISE). Additional parameters such as uric acid (UA), creatinine (Cr), alanine aminotransferase (ALT), aspartate aminotransferase (AST), troponin T, and creatine kinase MB (CK-MB) were analysed using an automated clinical chemistry analyser (e.g., Roche Cobas or Beckman Coulter AU series). Inflammatory and coagulation markers, including C-reactive protein (CRP) and D-dimers, were measured using a high-sensitivity immunoturbidimetric method. All biochemical and haematological tests were performed in a certified clinical laboratory following standard operating procedures to ensure precision and reliability.

#### 2.3.5. Blood Pressure

Blood pressure (BP) was measured in complete physical and mental calm. Patients assumed a sitting position for a minimum of 5 min before the measurement, and it was performed by nurses, who tested each respondent twice with a momentary pause between measurements, according to the recommendations of Frese et al. [22]. The average, which was calculated from the two measurements obtained, was used for the results.

### 2.4. Statistical Analysis

The results of the study were collected in an MS Excel spreadsheet, MS Office 2013, while the analysis was performed in Statistica 2013, Stat Soft Poland. Data were divided into measurable and non-measurable. The former were presented by: X (mean) and SD (standard deviation), as well as M (median) and Rk (interquartile range). If the distribution of the outcome data was symmetric, the mean was suggested, while if it was asymmetric, the median was used, and the Shapiro–Wilk test was used to evaluate this distribution. The analysis of differences depended on the shape of the distribution and the size of the group (two groups—Student’s *t*-test or Mann–Whitney U test was used, three or more groups—ANOVA variance or Kruskal–Wallis test, multiple groups—post hoc tests). Unmeasured data were presented as percentages and used χ^2^ tests of independence with corrections to the test (Yul’s test, Fischer’s test) depending on the expected numbers. The gamma correlation coefficient and Cramer’s V were also determined. Cochran’s Q test was performed to determine the concordance of the number of correct responses to the author’s questionnaires. The risk of metabolic syndrome was assessed by logistic regression analysis, for which NRS scale, age and blood tests were used, and the significance level was *p* < 0.05.

## 3. Results

### 3.1. Characteristics of the Study Group

The table shows the detailed characteristics of the study group, consisting of 301 patients admitted to the Department of Cardiology. The analysed sample was slightly outnumbered by women (51.2%) compared to men (48.8%). The mean age of the subjects was 67 ± 11 years, with women being on average older than men (68.7 ± 11.6 vs. 65.2 ± 10.1). The group was heterogeneous in terms of age, with the largest representation of patients in the 70–79 years range (36.9%) and the smallest under 50 years of age (9.3%). Table 1 shows the exact results of the study.

The table shows the distribution of patients in terms of the presence of hypertension and atherosclerosis by gender and age groups. Overall, of the 301 patients, the vast majority (79.1%) had a diagnosis of hypertension or atherosclerosis, while only 20.9% of patients did not have these conditions. In the analysis by gender, no significant statistical differences in the prevalence of conditions were found (*p* > 0.05). By age group, significant differences (*p* < 0.01) were observed in the prevalence of hypertension or atherosclerosis. The prevalence of these conditions increased with age. In the group under 50 years, 46.5 per cent of patients had hypertension or atherosclerosis, while in the group 70–79 years, this value reached 92.8 per cent. Detailed information is presented in Table 2.

### 3.2. Nutritional Status

The nutritional status of the study group shows considerable heterogeneity in terms of BMI, with overweight and obesity being the predominant problems among patients. The mean BMI for the entire group was 28.7 kg/m^2^, indicating an average value in the overweight range. Both women (28.7 kg/m^2^) and men (29.4 kg/m^2^) had elevated BMI values.

Overweight was the most common problem, comprising 47.5% of the study group. Among men, overweight was more common (57.8%) than in women (37.7%). Grade I obesity affected 18.9% of patients, with a clear predominance in women (27.9% vs. 9.5% in men). Grade II obesity was found in 13.6% of patients, mainly in the female group (14.9%). The study group is characterised by high rates of overweight and obesity, which increase with age, especially among those aged 60–79 years. Table 3 shows the exact anthropometric results of the studied group of patients.

The NRS 2002 scale was used to assess the risk of malnutrition. Overall, 13.6% of patients were found to be at no risk of malnutrition (NRS = 0), while low risk (NRS = 1) was present in 40.5% of participants. Moderate risk of malnutrition (NRS = 2) was present in 33.9% of participants, while high risk (NRS = 3 and NRS = 4) was found in 7% and 5% of patients, respectively. The results showed no significant differences between genders (*p* = 0.85370). An increase in the risk of malnutrition was observed with age but the relationship was not statistically significant (*p* = 0.54238) (Table 4).

### 3.3. Nutrition Knowledge

Analysis of respondents’ nutritional knowledge revealed statistically significant differences and areas with the highest and lowest levels of knowledge. In the area of number of meals per day, 4–5 meals were correctly indicated by 60.5% of respondents, with those with cardiovascular disease showing significantly higher knowledge (63.6%) compared to those without these conditions (48.4%; *p* < 0.0001). Similarly, greater knowledge occurred in the area of calcium sources, with 73.2% of those with cardiovascular disease indicating milk and dairy products as the best source of calcium, compared to 56.5% in the healthy group (*p* = 0.0153).

Significant differences were also observed in knowledge of sources of iron, with 40.6% of those with cardiovascular disease correctly identifying meat products as the best source compared to only 16.1% of those without these conditions (*p* < 0.0001). Knowledge of the effects of excess potassium in the diet was also important; 59.4% of those with cardiovascular disease knew that it could cause cardiac arrhythmias, compared to 46.8% in the healthy group (*p* = 0.0248). These differences indicate a higher level of knowledge among patients with cardiac problems.

The highest level of knowledge in the entire study group concerned the sources of fats, with 81.1% of respondents correctly identifying vegetable oils and marine fish as the healthiest sources of fat. An equally high percentage of respondents (76.1%) identified meat, fish and eggs as the best sources of dietary protein.

The areas with the lowest levels of knowledge were protein function and phosphorus sources. Only 31.9% of participants knew that the primary role of protein was a building function, with this knowledge being significantly lower in the group without cardiovascular disease (27.4%; *p* < 0.001). An even lower level of knowledge was noted in the area of phosphorus sources, with only 12% of respondents identifying cottage cheese and egg white as the best sources of phosphorus (Table 5).

**Figure 1:**
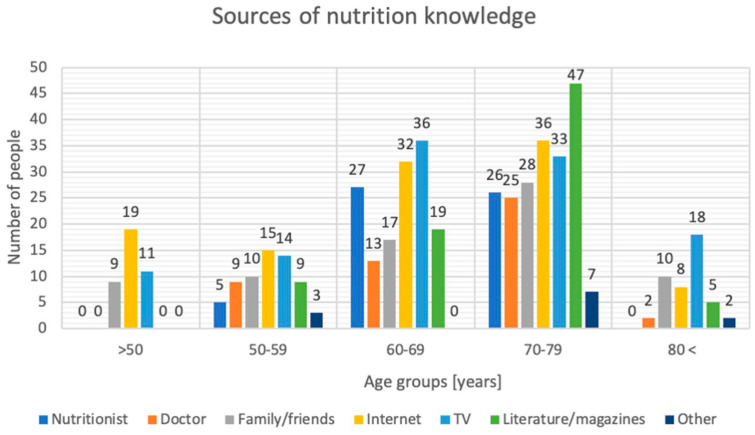
Sources of nutritional knowledge.

Respondents were asked where they got their knowledge about the principles of proper nutrition. Surprisingly, men were more likely to choose a nutritionist as a source (*p* = 0.005), while women were far more likely to use magazines and available literature (*p* = 0.001). When broken down by age, it can be seen that a nutritionist was most often chosen by those aged 60–69, while the older group of patients chose a doctor as their primary source of nutritional knowledge. In the youngest age group, those under 50 years of age, the Internet was the primary source of knowledge, chosen by as many as 67.9% of respondents (*p* = 0.01). The exact results are shown in Figure 1.

### 3.4. Eating Habits

The results of the eating habits survey showed that the respondents do not eat properly and their habits are inadequate. Important to note, and worrying, is the fact that only 9.3% of respondents, including only 8.8% with cardiovascular disease and 11.3% without cardiovascular disease, choose water as a beverage with their meals. The vast majority of respondents drink tea or juices with their meals. Among the statistically significant results, the fact that 83.4% of the respondents, including 90% of those diagnosed with cardiovascular diseases, consume the first breakfast is commendable. Patients overwhelmingly (72.4%, including 75.3% of those with cardiovascular disease) avoid snacking at night, but as many as 84.7% of respondents declared that they snack between meals (Table 6).

The questionnaire on the frequency of consumption of selected foods was divided into eight sections: (1) cereals and cereal products; (2) milk and dairy products such as kefir, buttermilk, yoghurt, cheese and eggs; (3) meat, cold cuts and fish; (4) vegetables, which were also categorised into several groups, as well as grains; (5) fruits; (6) products that are sources of fat; (7) beverages; and (8) sugar, honey and sweets.

From the group of cereal products, wholemeal bread was the most frequently chosen bread during the week and day. Respondents were also more likely to reach for coarse cereals than fine cereals. Patients with cardiovascular disease were also more likely to choose fibre-rich products during the day. However, these results were not statistically significant.

Milk and natural dairy products were the most preferred choice several times a week, while eggs were consumed most frequently by respondents several times a month, but these results were also not statistically significant. Respondents overall and with and without cardiovascular disease consumed cheese most often several times a month.

Among meat and charcuterie products, respondents were most likely to consume high-quality charcuterie, poultry and red meat during the month, which was also the most common choice of respondents with diagnosed cardiovascular disease, but these results were not statistically significant. Lean fish species were most often chosen by respondents several times a month (52.2%, including 51.9% with cardiovascular disease).

Vegetables and fruits were divided into numerous subgroups. Vegetable groups studied were: cruciferous, leafy greens, yellow-fleshed, root crops, legumes, potatoes and seeds and nuts. Fruits were grouped into tropical fruits, citrus fruits, stone fruits, berries, and bananas, pears, and apples. Dried fruit and fruit preparations were also included in this group. Among the significant results, it should be noted that tomatoes were most often consumed several times a week (37.9% overall and 41.1% by respondents with cardiovascular disease) and daily (34.6% overall and 41.9% of those without diagnosed cardiovascular disease). Among fruits, significant results were the consumption of apples and pears (28.6%), kiwis and citrus (26.2%) and bananas (25.9%) several times a week. Apples and pears were the most preferred fruit by respondents with cardiovascular disease (23%).

The healthiest fats, i.e., vegetable oils, were chosen most frequently by respondents, and they consumed them several times a week (33.2%) or daily (29.9%). The recommended amounts of never or almost never consumption of butter, animal fats, cream and mayonnaise and dressings were chosen relatively rarely (3.3%, 28.6%, 6.6%, and 20.9%, respectively). Respondents were far more likely to declare consuming these products more than a few times a month. The amount of animal fat consumption was not statistically significant. The fats most frequently chosen by people with cardiovascular disease were oils (daily 28.6% and several times a week 32.2%).

Beverages were grouped into fruit nectars and juices, vegetable juices and fruit and vegetable juices, hot drinks, energy drinks, sweetened drinks, and alcohol broken down into subgroups. The most commonly consumed beverages were those drunk warm. Vegetable juices were the most popular choice, consumed several times a week (23.6%) and daily (6%). It is commendable that statistically significant respondents declared that they do not consume energy drinks (60.8% of respondents and 59.4% of those with cardiovascular disease). Among sweets, a significant result was the low consumption of ice cream and pudding by respondents (13%, 21% without cardiovascular disease and 10.9% with cardiovascular disease). Table 7 shows detailed results.

### 3.5. Biochemistry and Blood Pressure

In the analysis of the respondents’ biochemistry results, no statistically significant differences were found between the cardiovascular disease group and the group of healthy participants. All values for the parameters analysed, such as blood pressure, morphological parameters (leukocytes, erythrocytes, haemoglobin, haematocrit), liver enzymes (AspAT, ALT), and metabolic indices, including cholesterol, glucose, CRP and D-dimers, did not differ significantly between the groups. Table 8 shows the exact results.

## 4. Discussion

### 4.1. Nutritional Knowledge Among Patients with Cardiovascular Diseases

This study showed that patients diagnosed with atherosclerosis and/or hypertension have different knowledge, nutritional habits and nutritional status compared to those without hypertension and atherosclerosis. Patients with these conditions showed greater knowledge of key dietary elements, such as the importance of calcium intake, iron sources and the effects of excess potassium. This is due to the fact that a disease diagnosis can be an important factor in mobilising greater knowledge in this area. The highest level of knowledge was noted in the area of fats. More than 80% said that the healthiest sources of fat were marine fish and vegetable oils. The majority of respondents (more than 75%) said that meat, fish and eggs are the best sources of protein.

### 4.2. Comparative Findings with Previous Studies

However, particularly among those without hypertension or atherosclerosis, there was a lack of correct information about the function of protein, fibre and phosphorus sources. Only 12% of respondents indicated that egg white and cottage cheese were the main sources of phosphorus, while 31.9% correctly identified the building function of protein.

A study by Italian researchers analysed the level of awareness regarding major cardiovascular risk factors among patients following a recent acute coronary syndrome (ACS). They observed a low level of knowledge about the role of physical activity and healthy eating habits as protective elements against cardiovascular disease. The results indicate a lack of awareness of the importance of physical activity as a protective factor against CVD in this group of patients. In addition, little knowledge of the role of smoking and diabetes as important risk factors for cardiovascular disease has been demonstrated [23].

Studies conducted among Polish patients have shown that the level of knowledge about food and nutrition was determined to be sufficient and dependent on the educational level of the respondents. It was pointed out that it is necessary to provide nutrition education on the correct nutritional principles for the disease, which will be tailored to individual patients after assessing their knowledge, eating habits and nutritional status [24].

Analysis of eating habits showed that many people do not follow dietary recommendations. Although 83.4% of the respondents regularly consumed the first breakfast, only 9.3% declared that water was the main beverage for meals in their diet, most of them drinking juices or tea. In addition, almost 85% of the respondents admitted that they snacked between meals, indicating habits that promote obesity and overweight. On the positive side, more than 72% of those who participated in the survey followed dietary recommendations regarding the daily rhythm of meal consumption. These results indicate the need for further educational efforts to permanently increase knowledge and change eating habits.

#### Dietary Habits and Compliance with Recommendations

Analysis of nutritional status showed that many patients were overweight or obese. These results are consistent with the observed correlation between abnormal body weight and hypertension and atherosclerosis. The mean BMI in the study group was indicative of overweight, and first- and second-degree obesity together affected more than 30% of the participants. Most of the patients were classified in the low-risk or moderate-risk group, according to the malnutrition risk assessment carried out on the basis of the NRS 2002 scale. The risk of malnutrition increases with age, indicating the need to pay more attention to the nutrition of elderly people with cardiovascular disease.

Differences in fasting glucose levels, lipidograms and inflammatory marker results were not significant between patients diagnosed with and without cardiovascular disease. Nevertheless, statistically significant differences in knowledge of sources of iron, calcium and the effects of excess potassium indicate greater nutritional awareness among patients with hypertension and atherosclerosis.

Respondents overwhelmingly reported eating breakfast I (83.4% of respondents, including 90% of those diagnosed with cardiovascular disease). Patients overwhelmingly (75%) avoid snacking at night, however, overeating between meals was declared by as many as 84% of respondents. Similar results were obtained in other studies where about 80% of respondents from Zabrze and Szczecin admitted to snacking between meals [24,25]. Matsuzaki et al. assessed the lifestyle of patients with acute myocardial infarction. Among all patients, 81% had a habit of snacking between meals, while 50% had a habit of eating snacks daily [26].

From the group of cereal products, respondents most often chose wholemeal bread during the week and day, while groats of both coarse and fine grains were chosen only once or a few times a month.

A study conducted by Bronkowska et al. at the Department of Occupational Diseases and Hypertension at the Wrocław Clinical Hospital [27] showed that the majority of hypertensive patients prefer white bread—a total of 91% of those surveyed. Dark bread, including graham-type products, was declared to be consumed by only 29% of women with the disease. These studies indicate that hypertensive patients consume too few whole grain cereal products, and that their diets are more heavily based on zoonotic products [27].

Another study, by Krêzel et al. in a group of patients with cardiovascular disease, found insufficient dietary intake of dark bread and furthermore, found a lack of foods with protective properties in their diets [24]. According to the American Heart Association (AHA) guidelines, a healthy diet is one of the eight key elements of cardiovascular health identified by Life’s Essential 8. Preventive measures recommend diets rich in, among other things, whole grains, in addition to vegetables, fruits, legumes and whole, lean protein sources, while avoiding and/or limiting highly processed foods, trans fatty acid isomers, and foods rich in simple sugars [28].

Similarly, an unsatisfactory level of coarse-grain products was shown in a study assessing the diet of cardiac patients by Szczepanska et al. Consumption of whole-grain bread was most often declared at the level of several times a week or not at all (19% each). In the case of whole-grain cereal products, such as groats and pasta, the predominant response was to consume them several times a week (29%) [25].

Milk and dairy products are particularly important because they are a good source of high-quality protein and minerals (calcium, magnesium, potassium), in addition to vitamin D and B vitamins [29]. Milk and natural dairy products were most often chosen several times a week by respondents. Both total respondents and those with and without cardiovascular disease were most likely to include cheese in their diet several times a month.

Similar results were obtained in a study by Krêżel et al. half of the respondents consumed various types of dairy products several times a week, including whole and low-fat milk at similar levels [24].

Milk and milk products (including yoghurt, kefir, and buttermilk) are rich in milk fat, and this fat is categorised as saturated fatty acids and may increase the risk of cardiovascular disease, but the results of cohort studies and randomised controlled trials in meta-analyses conducted are inconclusive. These studies indicate, however, that consumption of milk fat (low-fat and high-fat dairy products) does not increase the risk of cardiovascular disease [30].

A study controlling the dietary habits of patients with coronary artery disease (CAD) by Khatun et al. found no association between daily fat-free milk intake and weekly intake of cheese/butter/oil and cottage cheese/yoghurt with CAD risk [31].

Nutrition plays a key role in the prevention of premature death and disability associated with cardiovascular disease (CVD). Accordingly, international guidelines strongly emphasise the importance of a healthy diet, while recommending limiting the consumption of processed meat and fats to effectively control CVD risk factors [32]. The results show that respondents were most likely to choose high-quality meats, poultry and red meat.

Similar results of white and red meat consumption were obtained in a study of post-MI patients, where the frequency of consumption of these products several times a week was shown to be 57% and 36%, respectively [25]. Bronkowska et al. [27] showed in their study that for 54% of hypertensive women and 36% of hypertensive men, the fat content of the aforementioned food products was of great importance when purchasing meat and meat products.

Fish and shellfish (collectively referred to as fish) are among the most intensively studied food groups for their role in the diet and prevention of non-communicable diseases, particularly cardiovascular disease (CVD). Their beneficial effects are attributed primarily to their high content of polyunsaturated fatty acids (PUFAs), such as eicosapentaenoic acid (EPA) and docosahexaenoic acid (DHA). Accordingly, dietary recommendations for CVD prevention unequivocally recommend eating fish once to twice a week also for its other nutrients (vitamin D, riboflavin, iodine, calcium, phosphorus, magnesium, potassium, zinc and iron) [33]. The study found insufficient consumption of this group of products. Fish was most often chosen by respondents several times a month (more than half of the respondents). The results obtained indicate the necessary education of cardiac patients on the nutritional value of this group of products in the presence of cardiovascular disease. The results of a pooled analysis from four cohort studies from 58 countries by Mohan et al. showed that higher fish consumption (about 2 servings per week, i.e., ≥175 g/week) is associated with a lower risk of major cardiovascular events and mortality among patients with pre-existing vascular disease [34].

An optimal diet to reduce the risk of cardiovascular disease (CVD) should be based on whole grains, fruits, vegetables, legumes, nuts, fish, poultry and a moderate intake of dairy and vegetable oils beneficial for heart health. Such diets can reduce CVD risk by up to a third [35].

Diets such as the Mediterranean, DASH and healthy plant-based diets focus on increasing intake of fruits, vegetables, legumes, nuts and whole grains. Studies indicate that diets rich in fruits and vegetables are associated with a lower risk of cardiovascular disease and a lower risk of mortality [28]. The results of the study testify to the inadequate provision of vegetables in the diet, the daily intake of total vegetables was found to be 5–11% (different vegetable groups). Tomatoes were shown to be consumed more frequently (daily 33% and several times a week 41% of the subjects). In addition, the consumption of legumes was unsatisfactory, with respondents most often indicating consumption 1 or several times a month (40% and 30% of respondents). Inadequate intake of these products was also found in a study by Szczepanska et al., with 52% of respondents declaring pulses to be part of their diet 1–3 times a month, while vegetables were consumed several times a week by 42% of patients [25]. Low consumption of vegetables and legumes was also shown by Mikulska et al. in a study of the diet of patients with cardiovascular disease [36].

Analysis of the results showed that within the fruit group, the most frequent consumption at the level of several times a week was for apples and pears (29%), kiwis and citrus (26%), and bananas (26%). The most preferred fruits by respondents with cardiovascular diseases were apples and pears (23%). The aforementioned fruits are available all year round, firmly rooted in Polish culinary tradition and inexpensive.

Adequate supply with the daily diet of the recommended servings of vegetables, fruits and legumes will ensure sufficient dietary fibre and polyphenols, which provides benefits to the gut microbiome. The biodiversity of the gut microbiome benefits blood pressure control, reduces metabolic syndrome and diabetes, lowers levels of atherosclerotic compounds, and ultimately lowers CVD rates [28].

The American Heart Association (AHA) recommends the use of liquid oils. Use extra virgin olive oil (EVOO), rich in monounsaturated fats. Unsaturated fats present in liquid vegetable oils, such as soybean oil, corn oil, safflower oil and sunflower oil, as well as walnuts and flaxseeds, have been shown to have beneficial effects on cardiovascular health, lowering LDL-C and total cholesterol. In contrast, tropical oils (e.g., coconut, palm), animal fats (e.g., butter, lard) and hydrogenated fats raise LDL-C and total cholesterol, which negatively affect cardiovascular health [32].

The healthiest fats, i.e., vegetable oils, were chosen most often by respondents and they consumed them several times a week or daily (33% and 30%). The recommended amounts of never or almost never consumption of butter, animal fats, cream and mayonnaise and dressings were chosen less frequently (3–28%). The most preferred fats by people with cardiovascular disease were oils (daily 28% and several times a week 32%). Similar results to our own were obtained by Pilska [37], who conducted a study on a group of people residing in Poland and showed that the most preferred fat among those surveyed was butter, canola oil, followed by margarine, olive oil and sunflower oil.

The analysis of beverage consumption included the types of these products. It showed that the most frequently consumed beverages were those prepared hot. Vegetable juices were the most popular choices, with a few times a week (23%) and daily (6%). Respondents also declared that they do not consume energy drinks (59%). Similar results were obtained by Krêżel et al. the most preferred beverage consumed by respondents was hot tea, while 88% of respondents never consumed energy drinks [24].

In the case of alcohol consumption, it was shown that 37% of respondents declared that they do not consume alcohol, similar results were obtained in other studies, where a similar group of people indicated that they do not consume alcohol at all [25]. Similar results regarding alcohol consumption have been shown in studies in patients with myocardial infarction [24].

### 4.3. Effect of Diet on Hypertension

The study results confirm the importance of healthy eating habits in the prevention and control of hypertension. In particular, it has been shown that a high intake of vegetables, fruits, whole grain products, and unsaturated fatty acids is associated with a lower risk of hypertension and better blood pressure control [38].

Our results indicate that patients with greater nutritional knowledge were more likely to report reducing their salt intake, which is consistent with the findings of the INTERSALT study, which demonstrated a strong correlation between sodium intake and blood pressure levels [39]. Similar results were obtained in the study by He et al., which confirmed that a reduction in sodium intake by 1 g per day resulted in an average decrease in systolic blood pressure by 2–3 mmHg. In our study, patients who reported lower salt consumption also more frequently adhered to other healthy dietary principles, which may indicate their greater health awareness [40].

Another important factor is diet structure and its impact on blood pressure control. The study by Sacks et al. clearly demonstrated that individuals following the DASH diet (Dietary Approaches to Stop Hypertension) achieved an average reduction in systolic blood pressure by 5.5 mmHg and diastolic blood pressure by 3.0 mmHg compared to the control group [41]. In our study, individuals with higher nutritional knowledge more often chose foods consistent with the DASH diet principles, which could have contributed to their better health outcomes. Similar conclusions can be drawn from the study by Blumenthal et al., which emphasised that dietary interventions based on DASH, combined with weight reduction, lead to significant improvements in hypertension management [42].

Our results are also consistent with research on the impact of fibre intake on blood pressure. The study by Streppel et al. showed that individuals consuming higher amounts of fibre had significantly lower systolic and diastolic blood pressure values, which is attributed to improved endothelial function and reduced oxidative stress [43]. In our analysis, patients with higher nutritional knowledge more frequently consumed fibre-rich foods, such as whole-grain bread, groats, and vegetables, which aligns with these findings.

### 4.4. Socioeconomic and Cultural Factors, Practical Implications for Clinical Practice and Public Health

One of the key aspects influencing dietary habits and nutritional knowledge is the broader socio-economic, educational, and cultural context. In our study, we attempted to account for these factors by collecting demographic data, including education level, place of residence, and age, which were considered in the statistical analysis. Educational background has been identified as a significant determinant of nutritional knowledge, with previous studies suggesting that individuals with higher education levels tend to have better awareness of dietary recommendations and healthier eating habits. Cultural factors also play a crucial role in shaping dietary choices. Traditional dietary patterns, availability of specific foods, and cultural attitudes toward nutrition may impact adherence to dietary recommendations. Although our study was conducted in a relatively homogeneous population, future research should incorporate a broader cultural perspective to better understand dietary behaviours in diverse populations.

Given these considerations, future interventions aimed at improving nutritional knowledge and dietary habits should be tailored to specific socio-economic and educational backgrounds. Public health initiatives should prioritise accessibility to nutritional education, particularly among lower-income and less-educated populations, to ensure equitable health improvements across different demographic groups.

The findings of this study highlight the need for targeted nutritional education and dietary interventions among cardiology patients, particularly those diagnosed with hypertension and atherosclerosis. The observed deficiencies in nutritional knowledge and unhealthy dietary habits emphasise the necessity of integrating nutrition counselling into routine cardiology care, with healthcare professionals collaborating to develop personalised dietary plans aligned with evidence-based recommendations.

### 4.5. Strengths and Weaknesses of the Study

The strength of the study is that it conducted a multifaceted analysis that included an assessment of the patients’ eating habits, dietary knowledge and nutritional status. Biochemical and anthropometric parameters were also included, allowing for a holistic approach to the health of the subjects. Standardised tools such as the NRS 2002 scale were used, which increases the reliability and reproducibility of the results obtained. This makes the results comparable with other epidemiological and clinical studies.

The study also has weaknesses. It was cross-sectional, meaning that it presents the condition of patients at a single point in time. Such a model does not allow for the assessment of long-term changes in eating habits or the impact of dietary interventions on patients’ health. Additionally, the questionnaires on dietary habits and nutritional knowledge were based on patients’ self-reports, which introduces the risk of recall bias and social desirability bias, potentially leading to inaccurate or inflated responses.

It is also worth noting that the survey was conducted at a single centre in Sosnowiec, which may limit its generalisability to other regions with different dietary habits and levels of access to health education. Future research should consider implementing a longitudinal study design and multi-centre participation to improve the validity and applicability of the findings.

Another limitation of the study is that it did not comprehensively assess certain potential confounders, such as detailed medication use, socioeconomic status, and physical activity levels. Furthermore, while the sample size was statistically justified, the study did not control for specific dietary variations or individual metabolic differences, which could influence the observed relationships between dietary knowledge and hypertension.

To address these limitations, future studies should incorporate more objective dietary assessment tools (e.g., food diaries or biomarkers) to reduce self-reporting bias and include long-term follow-ups to better understand causal relationships between diet and blood pressure regulation.

## 5. Conclusions

The results obtained in the study indicate that the vast majority of respondents were diagnosed with diseases such as hypertension and/or atherosclerosis, with prevalence increasing with age. A significant proportion of respondents were found to have above-normal body weight, highlighting the need for targeted dietary interventions to mitigate cardiovascular risk factors. Although no increased risk of malnutrition was observed using the NRS 2002 scale, irregularities in dietary habits were evident, particularly concerning inadequate consumption of vegetables, fruits, pulses, whole grains, nuts, dairy products, and fish. Additionally, the very low intake of water as a primary beverage is concerning. On a positive note, respondents reported low consumption of sweetened drinks, energy drinks, alcohol, and salty snacks, which is beneficial for cardiovascular disease prevention.

These findings emphasise the urgent need for structured and personalised nutrition education programs aimed at improving patients’ dietary knowledge and behaviours, particularly for those with hypertension and atherosclerosis. Implementing evidence-based dietary guidelines into clinical practice could help prevent disease progression and reduce complications such as myocardial infarction and stroke. Furthermore, these results support the integration of nutrition counselling as a standard component of cardiovascular disease management in healthcare settings.

From a policy perspective, these findings highlight the importance of public health campaigns and regulatory measures to promote healthy eating habits, particularly in older populations. Encouraging collaboration between healthcare providers, dietitians, and policymakers can facilitate the development of targeted interventions, such as subsidising access to healthier food options and incorporating dietary education into routine medical care. By prioritising these changes, it is possible to reduce the burden of cardiovascular diseases and improve population-wide health outcomes.

## Figures and Tables

**Table 1:** Characteristics of respondents by age and gender.

		Sex			Age				
		Total	Women	Men	>50	50–59	60–69	70–79	<80
Number of persons N (%)		301 (100)	154 (51.2)	147 (48.8)	28 (9.3)	38 (12.6)	90 (29.9)	111 (36.9)	34 (11.3)
Age [years] X ± SD		67 ± 11	68.7 ± 11.6	65.2 ± 10.1	43.5 ± 5.6	56 ± 2.1	64.7 ± 3.1	73.7 ± 2.5	82.7 ± 2
Body weight [kg] X ± SD		80.3 ± 14.7	75.2 ± 13	85.7 ± 14.5	82 ± 21	76.6 ± 16.6	84.5 ± 14.7	81.2 ± 11.4	69.5 ± 9.4
Height [cm] X ± SD		167.4 ± 9.6	161.3 ± 7	173.8 ± 7.4	175 ± 13.2	169.3 ± 5.1	168.4 ± 9.2	166 ± 9	161.2 ± 7.4
Education	Primary N (%)	15 (5)	11 (7.1)	4 (2.7)	3 (10.7)	0 (0)	6 (6.7)	4 (3.6)	2 (5.9)
	Secondary N (%)	155 (51.5)	69 (44.8)	86 (58.5)	7 (25)	15 (39.5)	62 (68.9)	61 (55)	10 (29.4)
	Vocational N (%)	72 (23.9)	40 (26)	32 (21.8)	6 (21.4)	17 (44.7)	16 (17.8)	20 (18)	13 (38.2)
	Higher N (%)	59 (19.6)	34 (22.1)	25 (17)	12 (42.9)	6 (15.8)	6 (6.7)	26 (23.4)	9 (26.5)

X = average; SD = standard deviation.

**Table 2:** Characteristics of the subjects including the presence of hypertension and atherosclerosis.

Number of Patients N (%)	Sex			*p*-Value	Age					*p*-Value
	Total N = 301 (100)	Women N = 154 (100)	Men N = 147 (100)		>50 N = 28 (100)	50–59 N = 38 (100)	60–69 N = 90 (100)	70–79 N = 111 (100)	<80 N = 34 (100)	
Number of patients without known hypertension or atherosclerosis N (%)	62 (20.9)	27 (17.5)	35 (23.8)	*p* = 0.23	15 (53.5)	9 (23.7)	23 (25.6)	8 (7.2)	7 (20.6)	*p* = 0.43
Number of patients with known hypertension or atherosclerosis N (%)	239 (79.1)	127 (82.5)	112 (76.2)	*p* = 0.17	13 (46.5)	29 (76.3)	67 (74.4)	103 (92.8)	27 (79.4)	*p* < 0.01 *
Hypertension (I10–I15, ICD-10)	200 (66.2)	107 (69.5)	93 (63.3)	*p* = 0.25	8 (28.6)	22 (57.9)	58 (64.4)	93 (83.8)	19 (55.9)	*p* < 0.001 *
Atherosclerosis (I70, ICD-10)	104 (34.4)	53 (34.4)	51 (34.7)	*p* = 0.96	7 (25)	15 (39.5)	20 (22.2)	48 (43.2)	14 (41.2)	*p* < 0.05 *

* *p* < 0.05.

**Table 3:** The results of anthropometric studies of the study.

Metric Data		Total	Sex		Age				
Anthropometric Data			Women	Men	>50	50–59	60–69	70–79	<80
Total N (%)		301 (100)	154 (51.2)	147 (48.8)	28 (9.3)	38 (12.6)	90 (29.9)	111 (36,9)	34 (11.3)
BMI [kg/m^2^]		28.7 ± 4.7 28.7 (5.6)	29 ± 4.9 29.4 (5.7)	28.3 ± 4.5 27.4 (4.1)	26.5 ± 4.9 26.3 (2.5)	26.7 ± 5.5 25.3 (7.8)	29.8 ± 4.9 29 (8)	29.5 ± 3.9 29.3 (4.4)	26.8 ± 3.3 26.7 (4.8)
BMI N (%)	Underweight	6 (2)	6 (3.9)	0 (0)	3 (10.7)	0 (0)	0 (0)	3 (2.7)	0 (0)
	Normal weight	54 (17.9)	24 (15.6)	30 (20.4)	5 (17.9)	14 (36.8)	17 (18.9)	5 (4.5)	13 (38.2)
	Overweight	143 (47.5)	58 (37.7)	85 (57.8)	17 (60.7)	17 (44.7)	34 (37.8)	62 (55.9)	13 (38.2)
	Obesity of the first degree	57 (18.9)	43 (27.9)	14 (9.5)	0 (0)	3 (7.9)	17 (18.9)	29 (26.1)	8 (23.5)
	Obesity of II degree	41 (13.6)	23 (14.9)	18 (12.2)	3 (10.7)	4 (10.5)	22 (24.4)	12 (10.8)	0 (0)

X—average, SD—standard deviation, Rk—range, M—median.

**Table 4:** Results of patients’ nutritional status assessment using the NRS scale.

Predictors		Total N = 301 (100)	NRS					*p*-Value
			NRS = 0 N = 41	NRS = 1 N = 122	NRS = 2 N = 102	NRS = 3 N = 21	NRS = 4 N = 15	
Sex	Women N = 154 (100)	154 (51.2)	22 (14.3)	66 (42.9)	48 (31.2)	10 (6.5)	8 (5.2)	*p* = 0.85370
	Men N = 147 (100)	147 (48.8)	19 (12.9)	56 (38.1)	54 (36.7)	11 (7.5)	7 (4.8)	
Age	>50 N = 28 (100)	28 (9.3)	4 (14.3)	9 (32.1)	13 (46.4)	2 (7.1)	0 (0)	*p* = 0.54238
	50–59 N = 38 (100)	38 (12.6)	4 (10.5)	21 (55.3)	9 (23.7)	2 (5.3)	2 (5.3)	
	60–69 N = 90 (100)	90 (29.9)	12 (13.3)	40 (44.4)	30 (33.3)	4 (4.4)	4 (4.4)	
	70–79 N = 111 (100)	111 (36.9)	14 (12.6)	39 (35.1)	42 (37.8)	10 (9)	6 (5.4)	
	>80 N = 34 (100)	34 (11.3)	7 (20.6)	13 (38.2)	8 (23.5)	3 (8.8)	3 (8.8)	
Hypertension or atherosclerosis	No N = 62 (100)	62 (20.6)	6 (9.7)	26 (41.9)	20 (32.3)	5 (8.1)	5 (8.1)	*p* = 0.64247
	Yes N = 239 (100)	239 (73.4)	35 (14.6)	96 (40.2)	82 (34.3)	16 (6.7)	10 (4.2)	

**Table 5:** Correct answers concerning nutritional knowledge of respondents.

Nutritional Knowledge		Total N = 301 (100)	Hypertension or Atherosclerosis		*p*-Value
			No N = 62 (100)	Yes N = 239 (100)	
Recommended number of meals per day:	4–5 meals	182 (60.5)	30 (48.4)	152 (63.6)	*p* < 0.0001
Recommended number of hours between meals:	3–4 h	211 (70.1)	41 (66.1)	170 (71.1)	*p* = 0.56385
Best type of heat treatment:	Cooking	190 (63.1)	37 (59.7)	153 (64)	*p* = 0.13311
Function that protein has in the body:	Building function	96 (31.9)	17 (27.4)	79 (33.1)	*p* = 0.00014 *
In which products you will find the most protein:	Meat, fish, eggs	229 (76.1)	51 (82.3)	178 (74.5)	*p* = 0.09961
In which products you will find the most fat:	Vegetable oils and marine fish	244 (81.1)	50 (80.6)	194 (81.2)	*p* = 0.69708
What is dietary fibre responsible for:	Regulating intestinal motility	137 (45.5)	28(45.2)	109 (45.6)	*p* = 04583 *
In which products we find the most dietary fibre:	Cereal products, vegetables	152 (50.5)	28 (45.2)	124 (51.9)	*p* = 0.25956
In which products we will find the most sodium:	Meat and processed meat products	61 (20.3)	18 (29)	43 (18)	*p* = 0.61551
In which products we find the most potassium:	Legumes	194 (64.5)	45 (72.6)	149 (62.3)	*p* = 0.00176 *
Excess potassium can lead to:	Heart rhythm disturbances	171 (56.8)	29 (46.8)	142 (59.4)	*p* = 0.02480 *
In which products we find the most phosphorus:	Cottage cheese and egg white	36 (12)	7 (11.3)	29 (12.1)	*p* = 0.53287
Adequate daily calcium intake prevents:	Osteoporosis	165 (54.8)	32 (51.6)	133 (55.6)	*p* = 0.20548
In which products we find the most calcium:	Milk and dairy products	210 (69.8)	35 (56.5)	175 (73.2)	*p* = 0.01535 *
Iron is responsible for:	Transporting oxygen in the body	161 (53.5)	33 (53.2)	128 (53.6)	*p* = 0.29532
In which products we find the most iron:	Meat products	107 (35.5)	10 (16.1)	97 (40.6)	*p* < 0.0001
Which vitamins are fat soluble:	A,D,E,K	82 (27.2)	16 (25.8)	66 (27.6)	*p* = 0.36328
Where we find the most vitamin D:	Sunlight	185 (61.5)	46 (74.2)	139 (58.2)	*p* = 0.04722 *
In which products we find the most vitamin C	Fruits and vegetables	197 (65.4)	30 (48.4)	167 (69.9)	*p* = 0.01224 *

* = *p* < 0.05.

**Table 6:** Correct answers regarding the eating habits of the respondents.

Eating Habits		Total N = 301 (100)	Hypertension or Atherosclerosis		*p*-Value
			No N = 62 (100)	Yes N = 239 (100)	
Does he have his first breakfast in the morning?	yes, always	251 (83.4)	36 (58.1)	215 (90)	*p* < 0.0001
What do you eat most often for your first breakfast?	a sandwich with cold meat and/or cheese and vegetables	97 (32.2)	24 (38.7)	73 (30.5)	*p* = 0.62377
Do you eat a second breakfast?	yes, always	79 (26.2)	15 (24.2)	64 (26.8)	*p* = 0.06198
What do you most often eat for your second breakfast?	Fruit	98 (44.3)	14 (35.9)	84 (46.2)	*p* = 0.09934
What do you most often drink with your meal?	still mineral water	28 (9.3)	7 (11.3)	21 (8.8)	*p* < 0.0001
How many meals does he/she eat in a day?	4–5 meals	132 (43.9)	23 (37.1)	109 (45.6)	*p* = 0.45222
Do you ever skip meals?	no, never	46 (15.3)	5 (8.1)	41 (17.2)	*p* = 0.01603 *
Do you ever skip meals at night?	no, never	218 (72.4)	38 (61.3)	180 (75.3)	*p* = 0.04406 *
Does he/she consider his/her diet healthy?	No	43 (14.3)	17 (27.4)	26 (10.9)	*p* = 0.00507 *

* = *p* < 0.05.

**Table 7:** Results of frequency of consumption of food products.

Eating Habits		Total N = 301 (100)	Hypertension or Atherosclerosis		*p*-Value
			No N = 62 (100)	Yes N = 239 (100)	
Cereal Products					
Wholemeal bread	2 to 3 times a week	79 (26.2)	18 (29)	61 (25.5)	*p* = 0.10238
	daily	80 (26.6)	11 (17.7)	69 (28.9)	
	several times a day	10 (3.3)	0 (0.0)	10 (4.2)	
Refined bread	never or hardly ever	32 (10.6)	7 (11.3)	25 (10.5)	*p* = 0.077747
	once a month/less often	32 (10.6)	10 (16.1)	22 (9.2)	
	2 to 3 times a month	101 (33.6)	13 (21)	88 (36.8)	
Coarse-grain cereals	2 to 3 times a month	173 (57.5)	35 (56.5)	138 (57.7)	*p* = 0.09762
	2 to 3 times a week	51 (16.9)	8 (12.5)	43 (18)	
	daily	2 (0.7)	2 (3.2)	0 (0)	
Fine groats	never or hardly ever	15 (5)	2 (3.2)	13 (5.4)	*p* = 0.07770
	once a month/less often	48 (15.9)	9 (14.5)	39 (16.3)	
	2 to 3 times a month	173 (57.5)	39 (61.3)	135 (56.5)	
Milk Products and Eggs					
Milk and natural dairy drinks	2 to 3 times a week	112 (37.2)	23 (37.1)	89 (37.2)	*p* = 0.17446
	daily	26 (8.6)	4 (6.5)	22 (9.2)	
	several times a day	3 (1)	0 (0)	3 (1.3)	
Sweetened milk drinks	never or hardly ever	79 (26.2)	18 (29)	61 (25.5)	*p* = 0.71107
	once a month/less often	84 (27.9)	15 (24.2)	69 (28.9)	
	2 to 3 times a month	100 (33.2)	21 (33.9)	79 (33.1)	
Cheeses	never or hardly ever	13 (4.3)	4 (6.5)	9 (3.8)	*p* = 0.02255 *
	once a month/less often	72 (23.9)	11 (17.7)	61 (25.5)	
	2 to 3 times a month	115 (38.2)	27 (43.5)	88 (36.8)	
Eggs, egg dishes	2 to 3 times a month	166 (55.1)	37 (59.7)	129 (54)	*p* = 0.30049
	2 to 3 times a week	71 (23.6)	15 (24.2)	56 (23.4)	
Meat Products and Fish					
Sausages	never or hardly ever	22 (7.3)	4 (6.5)	18 (7.5)	*p* = 0.05149
	once a month/less often	33 (11)	7 (11.3)	26 (10.6)	
Premium cured meats	once a month/less often	7 (2.3)	2 (3.2)	5 (2.1)	*p* = 0.06358
	2 to 3 times a month	135 (44.9)	36 (58.1)	99 (41.4)	
	2 to 3 times a week	124 (41.2)	21 (33.9)	103 (43.1)	
Sausage products and offal meat	never or hardly ever	36 (12)	12 (19.7)	24 (10)	*p* = 0.29606
	once a month/less often	92 (30.6)	15 (24.2)	77 (32.2)	
	2 to 3 times a month	121 (40.2)	25 (40.3)	96 (40.2)	
Red meat	never or hardly ever	23 (7.6)	6 (9.7)	17 (7.1)	*p* = 0.17654
	once a month/less often	71 (23.6)	15 (24.2)	77 (32.2)	
	2 to 3 times a month	132 (43.9)	21 (33.9)	111 (46/4)	
Poultry meat and rabbit meat	2 to 3 times a month	142 (47.2)	29 (46.8)	113 (47.3)	*p* = 0.19000
	2 to 3 times a week	112 (37.2)	26 (41.9)	86 (36)	
Fish, lean	2 to 3 times a month	157 (52.2)	33 (53.2)	124 (51.9)	*p* = 0.01317 *
	2 to 3 times a week	50 (16.6)	16 (25.8)	34 (14.2)	
Fish, fatty	2 to 3 times a month	125 (41.5)	25 (40.3)	100 (41.8)	*p* = 0.12319
	2 to 3 times a week	59 (19.6)	17 (27.4)	42 (17.6)	
Vegetables And Grains					
Cruciferous vegetables (including cabbage, broccoli, cauliflower, kohlrabi, brussels sprouts)	2 to 3 times a week	110 (36.5)	24 (38.7)	86 (36)	*p* = 0.15360
	daily	20 (6.6)	4 (6.5))	16 (6.7)	
Yellow-orange vegetables	2 to 3 times a week	120 (39.9)	25 (40.3)	95 (39.7)	*p* = 0.46580
	daily	32 (10.6)	5 (8.1)	27 (11.3)	
	several times a day	3 (1)	0 (0)	3 (1.3)	
Leafy green vegetables	2 to 3 times a week	112 (37.2)	27 (43.5)	85 (35.6)	*p* = 0.75292
	daily	16 (5.3)	3 (4.8)	13 (5.4)	
	several times a day	3 (1)	0 (0)	3 (1.3)	
Tomatoes	2 to 3 times a week	114 (37.9)	15 (24.2)	99 (41.4)	*p* = 0.02319 *
	daily	104 (34.6)	26 (41.9)	78 (32.6)	
	several times a day	16 (5.3)	1 (1.6)	15 (6.3)	
Cucumbers	2 to 3 times a week	160 (53.2)	25 (40.3)	135 (56.5)	*p* = 0.13872
	daily	34 (11.3)	11 (17.7)	23 (9.6)	
	several times a day	8 (2.7)	2 (3.2)	6 (2.5)	
Root vegetables	2 to 3 times a week	126 (41.9)	33 (53.2)	93 (39.8)	*p* = 0.17444
	daily	26 (8.6)	4 (6.5)	22 (9.2)	
	several times a day	5 (1.7)	1 (1.6)	4 (1.7)	
Dried pulses	once a month/less often	127 (42.5)	30 (48.4)	97 (40.9)	*p* = 0.37525
	2 to 3 times a month	87 (29.1)	14 (22.6)	73 (30.8)	
Potatoes	2 to 3 times a week	79 (26.2)	16 (25.8)	63 (26.4)	*p* = 0.68117
	daily	78 (25.9)	20 (32.3)	58 (24.3)	
Nuts	2 to 3 times a week	57 (18.9)	15 (24.2)	42 (17.6)	*p* = 0.15679
	daily	6 (2)	0 (0)	6 (2.5)	
	several times a day	6 (2)	2 (3.2)	4 (1.7)	
Grains	2 to 3 times a month	77 (25.6)	11 (17.7)	66 (27.6)	*p* = 0.27067
	2 to 3 times a week	37 (12.3)	11 (17.7)	26 (10.9)	
	daily	2 (0.7)	0 (0)	2 (0.8)	
Fruits					
Fruits with seeds	2 to 3 times a week	101 (33.6)	18 (29)	83 (34.7)	*p* = 0.46428
	daily	28 (9.3)	4 (6.5)	24 (10)	
Citrus fruits and kiwi	2 to 3 times a week	79 (26.2)	30 (48.4)	49 (20.5)	*p* < 0.001
	daily	26 (8.6)	7 (11.3)	19 (7.9)	
Tropical fruit (including mango, pineapple, watermelon, lychee)	2 to 3 times a week	36 (12)	14 (22.1)	22 (9.2)	*p* = 0.06032
	daily	6 (2)	1 (1.6)	5 (2.1)	
Berries fruits	2 to 3 times a week	97 (32.2)	24 (37.8)	73 (30.5)	*p* = 0.05072
	daily	9 (3)	0 (0)	9 (3.8)	
Bananas	2 to 3 times a week	78 (25.9)	30 (48.4)	48 (20.1)	*p* < 0.001
	daily	27 (9)	2 (3.2)	25 (10.5)	
Pears and apples	2 to 3 times a week	86 (28.6)	31 (50)	55 (23)	*p* = 0.00029 *
	daily	55 (18.3)	6 (9.7)	49 (20.5)	
Dried fruit	2 to 3 times a week	39 (13)	7 (11.3)	32 (13.4)	*p* = 0.79574
	daily	8 (2.7)	1 (1.6)	7 (2.9)	
Sweet processed fruit and candied fruit	never or hardly ever	51 (16.9)	8 (12.9)	43 (18)	*p* = 0.48829
	once a month/less often	91 (30.2)	17 (27.4)	74 (31)	
	2 to 3 times a month	94 (31.2)	23 (37.1)	71 (29.7)	
Fats					
Oil	2 to 3 times a week	100 (33.2)	23 (37.1)	77 (32.2)	*p* = 0.01580 *
	daily	90 (29.9)	21 (33.9)	69 (28.6)	
Butter	never or hardly ever	10 (3.3)	0 (0)	10 (4.2)	*p* = 0.00238 *
	once a month/less often	16 (5.3)	7 (11.3)	9 (3.8)	
	2 to 3 times a month	43 (14.3)	8 (12.9)	35 (14.6)	
Cream	never or hardly ever	20 (6.6)	5 (8.1)	15 (6.3)	*p* = 0.00026 *
	once a month/less often	47 (15.6)	9 (14.5)	38 (15.9)	
	2 to 3 times a month	76 (25.2)	26 (41.9)	50 (20.9)	
Animal fat	never or hardly ever	86 (28.6)	25 (40.3)	61 (25.5)	*p* = 0.11942
	once a month/less often	65 (21.6)	11 (17.7)	54 (22.6)	
Mayonnaise and dressings	never or hardly ever	63 (20.9)	15 (24.2)	48 (20.1)	*p* = 0.02164 *
	once a month/less often	80 (26.6)	8 (12.9)	72 (30.1)	
	2 to 3 times a month	84 (27.9)	23 (37.1)	61 (25.5)	
Drinks					
Nectars and fruit juices	2 to 3 times a month	103 (34.2)	24 (38.7)	79 (33.1)	*p* = 0.74250
	2 to 3 times a week	56 (18.6)	8 (12.9)	48 (20.1)	
Vegetable or fruit and vegetable juices	2 to 3 times a week	71 (23.6)	15 (24.2)	56 (23.4)	*p* = 0.01749 *
	daily	18 (6)	4 (6.5)	14 (5.9)	
Hot drinks	never or hardly ever	10 (3.3)	1 (1.6)	9 (3.8)	*p* = 0.43250
	once a month/less often	7 (2.3)	0 (0)	7 (2.9)	
	2 to 3 times a month	21 (7)	4 (6.5)	17 (7.1)	
Energy drinks	never or hardly ever	183 (60.8)	41 (66.1)	142 (59.4)	*p* = 0.00434 *
Sweetened beverages	never or hardly ever	148 (49.2)	30 (48.4)	118 (49.4)	*p* = 0.32869
Beers	never or hardly ever	113 (37.5)	26 (41.9)	87 (36.4)	*p* = 0.12295
	once a month/less often	60 (19.9)	13 (21)	47 (19.7)	
Vodka and spirits	never or hardly ever	122 (40.5)	33 (53.2)	89 (37.2)	*p* = 0.08413
Sweets					
Sugar	never or hardly ever	83 (27.6)	19 (30.6)	64 (26.8)	*p* = 0.32701
Honey	2 to 3 times a month	55 (18.3)	9 (14.5)	46 (19.2)	*p* = 0.87247
	2 to 3 times a week	87 (28.9)	17 (27.4)	70 (29.3)	
	daily	25 (8.3)	6 (9.7)	19 (7.9)	
Chocolate and chocolate candies	never or hardly ever	32 (10.6)	4 (6.5)	28 (11.7)	*p* = 0.43713
Non-chocolate candies	never or hardly ever	52 (17.3)	7 (11.3)	45 (18.8)	*p* = 0.22586
Cookies and cakes	never or hardly ever	14 (4.7)	2 (3.2)	12 (5)	*p* = 0.19996
Pudding and ice cream	never or hardly ever	39 (13)	13 (21)	26 (10.9)	*p* = 0.04694 *
Salty snacks	never or hardly ever	120 (39.9)	27 (43.5)	93 (38.9)	*p* = 0.15191

* = *p* < 0.05.

**Table 8:** Results of morphology and biochemical tests.

Biochemical Parameters of Blood	Total N = 301 (100)	Hypertension or Atherosclerosis		*p*-Value
		No N = 62 (100)	Yes N = 239 (100)	
SBP [mmHg]	140.8 ± 20 140 (20)	142.7 ± 18.8 150 (15)	140.4 ± 20.3 140 (20)	*p* = 0.195
DBP [mmHg]	79.7 ± 12 80 (20)	81.7 ± 13 80 (15)	79.2 ± 11.7 80 (20)	*p* = 0.178
WBC [tys./µL]	8.3 ± 3.1 7.8 (2.8)	8.1 ± 2.3 7.9 (2.4)	8.4 ± 3.3 7.7 (3)	*p* = 0.758
RBC [mln/µL]	4.6 ± 0.8 4.6 (0.6)	4.6 ± 0.6 4.6 (0.6)	4.6 ± 0.8 4.6 (0.6)	*p* = 0.464
Hgb [g/dL]	17 ± 18.9 13.8 (1.8)	18.8 ± 24.3 13.9 (1.7)	16.5 ± 17.3 13.8 (2)	*p* = 0.586
Hct [%]	40.6 ± 4.3 41 (5.1)	40.8 ± 4.4 41.8 (5)	40.6 ± 4.2 41 (5.1)	*p* = 0.568
PLT [tys./mm^3^]	228.2 ± 73 220 (74)	228 ± 79.7 223 (81)	228.3 ± 71.3 220 (75)	*p* = 0.846
Na [mmol/L]	138.3 ± 3.7 138 (3)	138.6 ± 3.4 138 (3)	138.2 ± 3.8 138 (4)	*p* = 0.342
K [mmol/L]	4.3 ± 0.5 4.2 (0.5)	4.3 ± 0.5 4.3 (0.6)	4.2 ± 0.4 4.2 (0.5)	*p* = 0.449
AST [IU/L]	30 ± 13.1 26 (13)	31.7 ± 13.7 30 (15)	29.5 ± 13 25 (11)	*p* = 0.081
ALT [IU/L]	26.4 ± 14 23 (14)	27.1 ± 13.7 23 (18)	26.2 ± 14 23 (14)	*p* = 0.543
CK-MB [ng/mL]	16.9 ± 10.3 14 (8)	15.8 ± 8.5 13.5 (6)	17.1 ± 10.7 14 (8)	*p* = 0.482
Troponin T [µg/L]	1415.1 ± 6266.3 9.6 (126.7)	1391.9 ± 6115.6 9.2 (146.2)	1421.1 ± 6317.4 10.3 (126.7)	*p* = 0.893
TC [mg/dL]	186.4 ± 52 179 (62)	191.8 ± 56.5 190.5 (83)	185 ± 50.8 177 (61)	*p* = 0.380
HDL—C [mg/dL]	56.5 ± 18.3 54 (23)	53.1 ± 16.2 49 (21)	57.4 ± 18.7 54 (23)	*p* = 0.139
LDL—C [mg/dL]	115.9 ± 38.1 107 (50)	125.4 ± 43.1 119.5 (65)	113.5 ± 36.4 107 (46)	*p* = 0.067
TG [mg/dL]	143.8 ± 154.8 116 (62)	156 ± 127.8 124.5 (84)	140.6 ± 161.1 112 (59)	*p* = 0.067
Cr [mg/dL]	1.2 ± 1.2 1 (0.4)	1.4 ± 1.7 1.1 (0.4)	1.2 ± 1 1 (0.4)	*p* = 0.517
FC [mg/dL]	122.8 ± 49.6 109 (30)	125.2 ± 48.6 110 (28)	122.1 ± 49.9 109 (30)	*p* = 0.456
CRP [mg/L]	14.6 ± 37.8 4.5 (6.6)	8.5 ± 15.4 4.6 (4.6)	16.2 ± 41.6 4.5 (6.9)	*p* = 0.533
UA [mg/dL]	6.2 ± 2 5.9 (2.4)	6.2 ± 2.4 5.9 (2.8)	6.2 ± 1.9 5.9 (2.1)	*p* = 0.977
D-Dimery [ng FEU/mL]	2.5 ± 3.7 0.8 (3.4)	2 ± 2.2 1.3 (2.6)	2.7 ± 4.1 0.8 (3.8)	*p* = 0.740

X = average, SD = standard deviation; SBP—Systolic Blood Pressure; DBP—Diastolic Blood Pressure; WBC—White Blood Cells; RBC—Red Blood Cells; Hgb—Hemoglobin; Hct—Hematocrit; PLT—Platelets; Na—Sodium; K—Potassium; AST—Aspartate Aminotransferase; ALT—Alanine Aminotransferase; CK-MB—Creatine Kinase MB; Troponin T—Cardiac Troponin T; TC—Total Cholesterol; HDL-C—High-Density Lipoprotein Cholesterol; LDL-C—Low-Density Lipoprotein Cholesterol; TG—Triglycerides; Cr—Creatinine; FC—Fasting Glucose; CRP—C-Reactive Protein; UA—Uric Acid; D-Dimery—D-Dimers

## Data Availability

The original contributions presented in this study are included in the article. Further inquiries can be directed to the corresponding author.
